# Long‐term acclimatization to high‐altitude hypoxia modifies interhemispheric functional and structural connectivity in the adult brain

**DOI:** 10.1002/brb3.512

**Published:** 2016-06-27

**Authors:** Ji Chen, Jinqiang Li, Qiaoqing Han, Jianzhong Lin, Tianhe Yang, Ziqian Chen, Jiaxing Zhang

**Affiliations:** ^1^ Department of Medical ImagingFuzhou Dongfang HospitalXiamen UniversityFuzhouFujianChina; ^2^ Department of Physiology and NeurobiologyMedical College of Xiamen UniversityXiamenFujianChina; ^3^ Department of Clinical PsychologyGulangyu Sanatorium of PLAXiamenFujianChina; ^4^ Magnetic Resonance CenterThe Affiliated Zhongshan Hospital of Xiamen UniversityXiamenFujianChina

**Keywords:** Adult neuroplasticity, DTI tractography, high altitude, ICA‐based denoising, interhemispheric, resting‐state fMRI

## Abstract

**Background:**

Structural and functional networks can be reorganized to adjust to environmental pressures and physiologic changes in the adult brain, but such processes remain unclear in prolonged adaptation to high‐altitude (HA) hypoxia. This study aimed to characterize the interhemispheric functionally and structurally coupled modifications in the brains of adult HA immigrants.

**Methods:**

We performed resting‐state functional magnetic resonance imaging (fMRI) and diffusion tensor imaging (DTI) in 16 adults who had immigrated to the Qinghai‐Tibet Plateau (2300–4400 m) for 2 years and in 16 age‐matched sea‐level (SL) controls. A recently validated approach of voxel‐mirrored homotopic connectivity (VMHC) was employed to examine the interhemispheric resting‐state functional connectivity. Areas showing changed VMHC in HA immigrants were selected as regions of interest for follow‐up DTI tractography analysis. The fiber parameters of fractional anisotropy and fiber length were obtained. Cognitive and physiological assessments were made and correlated with the resulting image metrics.

**Results:**

Compared with SL controls, VMHC in the bilateral visual cortex was significantly increased in HA immigrants. The mean VMHC value extracted within the visual cortex was positively correlated with hemoglobin concentration. Moreover, the path length of the commissural fibers connecting homotopic visual areas was increased in HA immigrants, covarying positively with VMHC.

**Conclusions:**

These observations are the first to demonstrate interhemispheric functional and structural connectivity resilience in the adult brain after prolonged HA acclimatization independent of inherited and developmental effects, and the coupled modifications in the bilateral visual cortex indicate important neural compensatory mechanisms underlying visual dysfunction in physiologically well‐acclimatized HA immigrants. The study of human central adaptation to extreme environments promotes the understanding of our brain's capacity for survival.

## Introduction

The human brain has the lifelong ability to reorganize structural and functional networks to adjust to environmental pressures and physiologic changes (reviewed by Pascual‐Leone et al. 2005; Zatorre et al. 2012). Currently, as millions of low land residents move to high altitude (HA) areas due to immigration, work duty or tourism, understanding the central adaptive processes in response to the previously reported neurologic deficits and cognitive dysfunctions occurring in the acute ascent period (Virués‐Ortega et al. 2004; Wilson et al. 2009) are important. The most prominent and important impact of living in an HA environment is chronic hypoxia. In addition to brain hypometabolism (Hochachka et al. 1994; Richardson et al. 2011), neuroimaging studies in recent years have revealed compensatory processes for brain structure and function in indigenous residents and immigrants who were born and raised in HA (Yan et al. 2010, 2011a; Zhang et al. 2010; Wang et al. 2014). Moreover, the physiological adaptation during a long period of HA exposure, such as alterations in circulatory and respiratory function, may lead to cumulative changes in the brain through afferent feedback (Penaloza and Arias‐Stella 2007). However, to purely investigate the plasticity of the adult brain in adapting to an HA environment, findings from the majority of previous studies were perturbed either by genetic regulation (Yan et al. 2010, 2011a), developmental experience of childhood, and adolescence (Zhang et al. 2010), or intermittent sea‐level (SL) normoxic exposure (Wang et al. 2014). Few studies, until now, have investigated the functional organization and underlying anatomic connectivity in adult HA immigrants who were born and raised in an SL area.

Brain hypometabolism has been proposed as a defense mechanism during HA acclimatization (Hochachka et al. 1996). Accordingly, decreased hemodynamic responses have been consistently reported in native HA residents during simple visual stimulation and the stimuli involved in sensory (visual information) input (Yan et al. 2011a,b). Unlike the task‐invoked fMRI, BOLD‐fMRI signal fluctuations at resting state reflect the intrinsic functional organization and endogenous neurophysiological processes of the human brain (Fox et al. 2005). Using resting‐state fMRI, Yan et al. (2010) observed that regional synchrony of spontaneous neural activity was increased in frontal and lingual cortices in adults born and raised at HA. With the same postprocessing method, increased regional homogeneity of base‐line neural activity in sensorimotor cortex was found in adult HA immigrants (Chen et al. 2016). Comparatively, one resting‐state electroencephalography study reported that the amplitudes of the delta and beta frequencies were reduced in adolescents living at HA (Richardson et al. 2011). Furthermore, it has been suggested that brain cellular structures are more inclined to integrate into highly dynamic and plastic neural networks when adapting to environmental and internal changes (Muotri and Gage 2006). All the aforementioned events indicate that strengthening the temporal synchronization of spontaneous neuronal activity (i.e., functional connectivity) may be involved as a compensatory mechanism in the adult brain during prolonged HA acclimatization.

Resting‐state functional homotopy is one of the most salient aspects of the brain's intrinsic functional architecture that likely reflect the importance of interhemispheric communication to integrated brain function underlying coherent cognition and behavior (Salvador et al. 2008; Stark et al. 2008). Interhemispheric interaction is vital for seamless representation of both halves of the body, particularly for the human visual system (Berlucchi 2014). Although the visual cortex and visuospatial function are vulnerable in the cohort of HA immigrants (Virués‐Ortega et al. 2004), a recent event‐related potential study demonstrated that the adult brain could employ hemispheric compensation during discrimination processes in early spatial attention processing stages after long‐term HA adaptation (Wang et al. 2014). Furthermore, functional connectivity and structural connectivity inherently overlap. It has been demonstrated that resting‐state functional connectivity reflects the structural connectivity architecture, and changed anatomical connectivity is related to an alteration in spontaneous BOLD correlations (van den Heuvel et al. 2009; Honey et al. 2009). Previous studies revealed that the corticospinal tract, as well as the corpus callosum (anterior and posterior body, splenium) (CC) are particularly susceptible to hypoxia (Zhang et al. 2012, 2013; Hunt et al. 2013). Furthermore, increases in FA values in the corticospinal tract and CC were only found in HA immigrant descendants (Zhang et al. 2010). This hypoxia‐adaptive genetic trait of callosal fiber modification indicates that CC may play a crucial role in brain anatomical architecture during HA acclimatization. The CC is a primary means for information transmission between the two hemispheres, and a previous study found that a high proportion of callosal fibers were dedicated to homotopic connectivity (Innocenti 1986). Therefore, interactive modifications of interhemispheric functional and structural connectivity were examined in this study. Changes in midline fusion regions, especially for the visual cortex, were predicted.

In this study, 16 SL natives who have immigrated to the Qinghai‐Tibet Plateau (2300–4400 m) for 2 years were recruited. They were all well‐acclimatized to the HA environment. A recently validated approach, resting‐state voxel‐mirrored homotopic functional connectivity (VMHC), was employed to investigate interhemisphere homotopic connections (Zuo et al. 2010). VMHC quantifies the resting‐state functional connectivity between each voxel in one hemisphere and its mirrored counterpart in the opposite hemisphere, and the method adopted here has proved to be informative in a diverse array of clinical studies (e.g., Anderson et al. 2010). Deterministic DTI tractography was adopted to quantify the anatomical connectivity corresponding to the changed homotopic functional connectivity. We hypothesized that interhemispheric functional connectivity (as measured by VMHC) could be modified during prolonged HA adaptation in the adult brain and that alterations to anatomical connectivity corresponding to the changed functional connectivity could be uncovered.

## Materials and Methods

### Subjects

Sixteen healthy male soldiers who had immigrated to the Qinghai‐Tibet Plateau (2300–4400 m) for 2 years were studied (Table 1). They were originally native lowlanders born and living at SL (Fujian Province) before immigrating to HA. They had no chronic mountain sickness. According to previous studies, a permanent and stable acclimatization to 3500 m is achieved in approximately 4 weeks, and complete hematocrit adaptation is achieved after 40 days (Zubieta‐Calleja et al. 2007). In this regard, 2 years is adequate time for the human body to adapt to HA through long‐term acclimatization. Sixteen male control subjects of comparable age and educational background were recruited in Xiamen (100 m). All subjects were from the Han population to avoid possible ethnic and ancestral differences. They were native lowlanders from the same SL places (Fujian Province) as the HA immigrant soldiers, and had left their hometown for 2 years. All subjects were right‐handed nonsmokers with normal body weight and body mass index, and had no documented neurological disorders or history of head injuries with loss of consciousness. The experimental protocol was approved by the Ethical Committee of Xiamen University. Before the experiments, the subjects were informed of the objectives, requirements, and procedures of the experiments, and all subjects provided written informed consent that has been archived. Subjects were compensated for participation.

**Table 1:** Demographic and physiological characteristics of HA immigrants and control subjects

Characteristics	HA immigrants (*n* = 16)	Controls (*n* = 16)	*P*	*d*	Minimum *N*
Demographic					
Age (years)	20.5 ± 0.7	19.9 ± 1.5	0.171		
Weight	60.1 ± 5.0	58.9 ± 5.2	0.456		
Education (years)	6.7 ± 3.9	7.5 ± 5.0	0.590		
Blood					
RBC (10^12^/L)	5.6 ± 1.0	4.7 ± 0.3	**0.007**	1.22	12
HGB (g/L)	159.8 ± 11.4	140.9 ± 8.1	**<0.001**	1.91	6
Pulmonary function					
VC (% predicted)	92.7 ± 14.9	103.2 ± 7.7	**0.007**	−0.89	21
FVC (% predicted)	84.1 ± 15.8	100.0 ± 10.3	**0.001**	−1.19	13
FEV1 (% predicted)	78.2 ± 14.6	96.5 ± 15.5	**0.001**	−1.22	12
FEV25%	4.8 ± 1.4	6.2 ± 1.4	**0.004**	−1.00	17
FEV50%	4.1 ± 1.1	4.4 ± 1.0	0.412	−0.29	188
FEV75%	2.9 ± 0.9	2.2 ± 0.6	**0.013**	0.92	20

VC, vital capacity; FEV, forced expiratory volume; FVC, forced vital capacity; HGB, hemoglobin; RBC, red blood cell.

The *P*‐values in bold indicate a significance of *P *<* *0.05. Effect size (*d*) was computed according to the formula presented by Cohen. The minimum *N* refers to the equal number of subjects in each group we need to recruit to detect a significant change at the 0.05 level corresponding to a power of 0.8 and the effect sizes (*d*) we reported here.

Unless otherwise indicated, the data are given as the means ± standard deviation.

### Physiological and neuropsychological tests

After HA immigrants gradually descended to SL for MRI scanning, subjects underwent physiological measures and neuropsychological tests in Zhongshan Hospital, Xiamen, Fujian province, China, within 7 days. Physiological tests include arterial blood pressure measures, arterial blood gas analysis, and pulmonary function measures. Blood samples were taken in the morning between 07:00 and 07:30 h. Several neuropsychological tests were administered with the details presented below.

(1) In the number search test, we presented any 8 of 9 numbers (range from 1 to 9) to the subjects each time, and the subjects were instructed to figure out the missing number as soon as possible. This test is thought to reflect speed of visual processing (Wei et al. 2007). (2) The visual reproduction and digit span tasks we performed were taken from the Chinese revised version of the Wechsler Memory Scale and were administered in line with our previous studies (Zhang et al. 2013; Chen et al. 2015). In the visual reproduction test, the participant is asked to reconstruct four geometric designs on paper by hand drawing after they observed each design for 10 sec (immediate recall). The digit span test requires the examiner to verbally present digits at a rate of one per second. (3) The Rey‐Osterrieth Complex Figure (ROCF) task was employed to assess the short‐ and long‐term visual memory and visuospatial construction ability. During the ROCF test, the subjects were told to draw a given complex figure with pens in four colors. The colors were changed in a fixed order so that the examiner could track the drawing sequence. Immediately following completion of the copy trial, the figure was removed and subjects were asked to reproduce the figure from memory (testing for immediate recall), and then the subjects were asked to reproduce the figure from memory again (delayed recall) after approximately 20 min. (4) The memory search task was adopted to evaluate the performance of retrieval from long‐term memory. The examiners asked the subjects to retrieve a previous event that was related to the cue words, and then the reaction time was recorded. The cue words we used were taken from the multimedia system of neuropsychological measurements that have been described elsewhere (Hu et al. 1999). (5) The mental rotation task we employed was a version of the task described in Gong et al. (2009). This mental rotation paradigm detects both spatial orientation ability and spatial visualization. Specifically, we used English capital letter “R” as the stimulator and included six different angles (range from 0° to 300° with an interval of 60°) and two different orientations (forward direction and mirrored) of “R” letters. During the experiment, these 12 “R” pictures emerged sequentially on the screen, and the subjects were asked to judge whether they were forward direction “R” or mirrored “R”. Mood tests were also performed using the Self‐Rating Anxiety Scale and the Self‐Rating Depression Scale.

### MRI data acquisition

MRI data acquisition was performed on a SIEMENS 3.0 T scanner (Erlangen, Germany) at the magnetic center of Zhongshan Hospital. Four minutes of resting‐state function images were obtained using an echo‐planar imaging sequence with the following parameters: TR/TE = 3000 ms/30 ms, flip angle = 90°, matrix = 64 × 64, FOV = 24 × 24 cm^2^, slices = 38, and thickness = 3 mm. All subjects were instructed to lie still in the scanner with their eyes closed and to remain awake. A DTI pulse sequence with single shot diffusion‐weighted echo planar imaging (TR/TE = 11000/96 ms, FOV = 184 × 184 mm^2^, NEX = 1, matrix = 128 × 128, slice thickness = 3 mm) was applied sequentially in 30 non‐collinear directions (b‐value = 1000 sec/mm^2^) with one scan without diffusion weighting (b = 0 sec/mm^2^). We acquired 50 contiguous slices covering the whole brain.

### Functional images

#### Resting‐state data processing

Data preprocessing was performed using the various tools in FMRIB's Software Library (FSL, www.fmrib.ox.ac.uk/fsl; RRID: SCR_002823). The first four volumes for each subject were discarded to eliminate the effects of T1 relaxation signal equilibrium. The remaining images were corrected for interleaved slice acquisition, and rigid‐body head motion correction was then applied using FSL MCFLIRT (Jenkinson et al. 2002). Any subject who had a maximum displacement greater than 2 mm or 2 degrees in any of the six parameters (x, y, z, pitch, roll, yaw) was excluded. High‐pass temporal filtering (sigma 50 s) was performed to remove low‐frequency drifts. As noise removal was performed in the time‐domain (frequency band pass filtering; e.g., 0.01–0.08 Hz) and a model of the CSF (by regressing out the covariate of CSF signals), it may be difficult to completely remove effects of vascular components (such as cardiac pulsation and blood vessels) in a variation in signals in brain tissues (Lowe et al. 1998; Shmueli et al. 2007; Murphy et al. 2013). We adopted a recent validated approach of automatic denoising (FMRIB's independent component analysis (ICA)‐based X‐noiseifier; FIX) for data from each scan (Salimi‐Khorshidi et al. 2014). The T2* effect is a nonlinear phenomenon, and decomposing the data into a set of artifactual components and nonartifactual components (i.e., fluctuations of interest) and regressing out the artifactual components from the data are therefore particularly useful in the case of subjects whose baseline physiological statuses have changed (e.g., increased hemoglobin concentration in our HA cohorts) (Murphy et al. 2013). ICA has been demonstrated to be a powerful method to remove head movement, physiological and scanner sources of fluctuations that are isolated from the various components found in resting‐state fMRI data allowing further voxel‐by‐voxel correlation analysis (e.g., Beall and Lowe 2007). The recently developed automatic version of ICA‐based denoising (FIX) has shown good to excellent performance across various fMRI datasets against manual component classifications (Salimi‐Khorshidi et al. 2014). A brief description of FIX processing was given as follows. Before executing the FIX plugin, single‐subject ICA was used to decompose the preprocessed data into a set of independent components (Beckmann and Smith 2004), and then FIX could automatically classify the ICA components as “good” (for signal) or “bad” (for noise) components with the trained‐weights files. Here, the “Standard.RData” was chosen as the training dataset for it was close to our data acquisition parameters. The results of the automatically defined signal and noisy components were visually inspected to ensure the accuracy of classification. After changing the threshold of good versus bad components from 5 to 25, we found that a threshold of 20 worked best for our data. Finally, the time series of the structured noises (based on the unique variance related to the noise components and motion confounds from the preprocessed data sets) were regressed out of the original data (Griffanti et al. 2014). The noise‐removed functional images were spatially transformed to a common stereotaxic coordination for subsequent analysis. Each participant's T1 image was coregistered to the mean realigned functional images. The transformed structural images were segmented into GM, WM, and CSF using a unified segmentation algorithm and spatially normalized to Montreal Neurological Institute (MNI) space nonlinearly during the estimation procedures. These transformation parameters were applied to functional images that were then resampled to 2 mm isotropic voxels.

#### Voxel‐mirrored homotopic connectivity

To account for differences in the geometric configuration of the cerebral hemispheres, we refined the preprocessed four‐dimensional residual functional images to a symmetric space. Methods for symmetric template creation and VMHC computation were consistent with Zuo et al. (2010). All 31 normalized GM images were averaged to create a mean image, which was then averaged with its left–right mirror versions to generate a group‐specific, symmetrical, study‐specific GM template. Finally, the normalized GM images were registered to this symmetric template and nonlinear transformation parameters were applied to the normalized functional images. After these deformations, the refined functional images were spatially smoothed with a 6‐mm full‐width at half‐maximum isotropic Gaussian kernel preparing for subsequent VMHC computation. The VMHC computation was performed using REST software. For each subject, the homotopic resting‐state functional connectivity was computed as the Pearson correlation coefficient between each voxel's time series and that of its symmetrical interhemispheric counterpart. The correlation values were then Fisher Z‐transformed to improve normality. The resultant values constitute the VMHC and were used for group‐level analyses. The global VMHC was also calculated for each subject by averaging VMHC values in all brain voxels within a mask (there was only one correlation for each pair of homotopic voxels). The mask was unilateral hemispheric gray matter, which was generated by thresholding the above produced symmetric template at 0.25. The thresholded mask was also used for group‐level analysis.

For an accurate localized spatial mapping, we applied the inverse transformation to the significantly altered clusters to bring them from the symmetric space back to each subject's asymmetric normalized functional space. Then, we resampled these clusters into a standard subject (fsaverage) using the nearest‐neighborhood interpolated sphere‐registration method in FreeSurfer and projected the transformed clusters on the Population‐Average, Landmark‐ and Surface‐based Brodmann Area atlas (PALS‐B12) (Van Essen 2005).

### DTI

#### Data preprocessing and diffusion tensor parameter calculation

DTI data were preprocessed and analyzed using the FSL software packages (http://fsl.fmrib.ox.ac.uk/fsl) and the diffusion toolkit (http://trackvis.org/). All raw images for each subject from the diffusion sequence were resampled to 2 mm isotropic voxels. Then, the nonbrain tissue components and background noise of these diffusion tensor images were removed after correction for eddy‐current induced distortion and head motion. Subsequently, the diffusion tensor model was fitted at each voxel using a least‐squares algorithm. The eigenvalue decomposition of the diffusion tensor was computed. The FA was calculated as the normalized standard deviation of the three eigenvalues and indicated a quantitative index of the degree of anisotropy of biological tissues. The FA values vary from 0 to 1 and putatively reflect WM spatial organization and integrity (Basser and Pierpaoli 2011). Finally, the gradient direction of each DTI volume was rotated according to the resultant affine transformations (Leemans and Jones, 2009). Whole‐brain fiber tracking was performed using the diffusion toolkit in the DTI native space (FA map) for each subject with fiber assignment by continuous tracking algorithm. Path tracing proceeded until the FA fell below 0.15, or until the minimum angle between the current and previous path segments was higher than 40° in case of sharp turns in the fiber direction as in previous studies (e.g., Thomas et al. 2011). Fibers less than 20 mm or with obvious false paths were discarded.

#### Tractography

To test whether the functional changes were associated with corresponding alterations of anatomic connectivity, homotopic regions showing changed VMHC in HA immigrants were adopted as regions of interest (ROIs) for an analysis of DTI tractography. Before the fibers connecting bilateral ROIs were tracked, several procedures were performed to inverse the ROIs from symmetric functional space into native individual *B*0 space (diffusion space). (1) We transformed these ROIs from the normalized symmetric space to each individual's normalized, geometric, asymmetric functional space, and then to its corresponding native functional space. (2) We coregistered the mean realigned functional image (native functional space) to the *B*0 image and applied the transformation to all ROIs, and (3) we dilated the ROIs by one voxel into the WM to ensure that they were in contact with the fibers. Whole‐brain fiber construction was accomplished using TrackVis software (www.trackvis.org; RRID: SCR_004817), and the fiber bundles connecting each pair of ROIs in the two hemispheres were then extracted from the total collection of brain fibers. To determine through which part of the corpus callosum the commissural fibers pass, we normalized the tracts of all subjects from the individual *B*0 spaces to the MNI space. Firstly, we coregistered the T1 image to the *B*0 image, and then we normalized the coregistered T1 image to the MNI space and obtained the transformation matrices. After that, we extracted the commissural fibers connecting bilateral ROIs in individual *B*0 space and warped them to the MNI space using the transformation matrices defined above. Finally, a population‐based probabilistic map of commissural tracts was produced after adding up all the binarized normalized tracts.

The path length was calculated by averaging the lengths of all the fibers connecting the homotopic ROIs, which is a basic measure characterizing two connective nodes. The FA value was extracted from each corresponding modality of tensor maps within the binarized commissural fiber mask of each subject.

### Statistical analysis

#### Physiological and neuropsychological data

The physiological measurements and neuropsychological tests were compared between the two groups using two‐sample two‐tailed *t*‐tests. Statistical significance was set at *P *<* *0.05.

#### Functional MRI data

A two‐tailed, voxel‐wise *t*‐test design was used to test for regional differences in VMHC between the two groups, and demographic variables of age and education years were entered as covariates of no interest in the comparisons to derive the results. Multiple comparisons were corrected within one hemisphere (only one correlation for each pair of homotopic voxels) based on Gaussian random field (GRF) theory [minimum Z > 2.57 (two‐tailed), uncorrected; cluster level, *P *<* *0.05, corrected]. We also ran the statistical test again to control for the hematocrit level in the group‐level analysis. Although our previous morphometric study in this same sample did not reveal a significant between‐group difference in gray matter volume within the bilateral visual cortex, here we further performed an analysis to control for voxel gray matter volume in between‐group comparisons to eliminate the potential structural differences.

#### DTI data

The resulting values of FA and fiber length were compared between the two groups. Two‐sample *t*‐tests and nonparametric tests were used where appropriate depending on the distribution of the data. One‐sample Kolmogorov–Smirnov tests were used to evaluate distribution normalities. A Bonferroni correction was used to address the problem of multiple comparisons.

#### Correlations

Mean z‐VMHC value was extracted from each individual's normalized map within the clusters showing significant alterations in HA immigrants for subsequent correlation analysis. These averaged values were correlated with physiological indices, cognitive measurements, altitude, quadratic altitude, and cubic altitude using Pearson correlation analysis in SPSS (SPSS Inc., Chicago, IL). Due to the relatively large number of cognitive and physiological measurements involved, the results of the correlation analysis were adjusted with a Bonferroni correction to control for family‐wise error related to multiple tests (overall *α *< 0.05). For cognitive measurements, the correlation analysis was run again after controlling for age and education. To investigate the potential altitude‐sensitive regions, we performed a supplementary whole‐brain correlation analysis in REST. Altitude, quadratic altitude and cubic altitude was entered as covariates of interest, and then Pearson correlation coefficients were calculated between these altitude parameters and voxel z‐VMHC values. To control for multiple comparisons, the statistical whole‐brain correlation maps were corrected using GRF at the group‐level analysis (Z > 2.57, cluster level *P *<* *0.05, corrected).

The relationship between functional and structural connectivity was analyzed using Pearson or spearman correlation analysis in SPSS. Specifically, commissural fiber parameters (FA and fiber length) were correlated with the extracted mean z‐VMHC values. Subjects with reconstructed commissural fibers in each group were combined for this correlation analysis. Significant correlations corrected for family‐wise error rate using a Bonferroni correction (equivalent to an uncorrected *P* value of 0.025) are reported.

Given the relatively small sample size we recruited in this study, we further performed a power analysis using R software (https://www.R-project.org/; RRID: SCR_001905) to complement the significant tests.

### Additional analysis of spatial and time‐series signal‐to‐noise

The BOLD effects of interest are only a few percent in magnitude, and these expected signal changes originate from neural activity that is sensitively influenced by the intrinsic image time‐series fluctuation levels (Friedman and Glover 2006). Physiologic adaptation in HA immigrants, especially increased hemoglobin content (coupled with decreased blood oxygen saturation), may affect the BOLD signal, which may potentially contribute to the resulting fMRI and tractographic metrics. For this reason, in addition to applying FIX‐based denoising, we implemented a confirmatory analysis to calculate the spatial SNR (sSNR) for *B*0 images of diffusion MR imaging (dMRI) and time‐series SNR (tSNR) of fMRI data to determine whether our results reflect neural activity or merely vascular effects. tSNR was used to evaluate the temporal stability of the measured time course of the resting‐state fMRI data. sSNR was calculated as the ratio of the mean signal to the standard deviation of the background noise (Song et al. 2015). tSNR was estimated as the ratio of the mean signal from all the voxels within the ROI, averaged across time and then divided by the standard deviation across time (Parrish et al. 2000). The standard deviation of background noise was measured using two 4 × 50 rectangular ROIs located at bilateral edges of the image (avoid ghosting/aliasing or motion artifact regions) in the central slice. The measurements of the objective mean signal were evaluated within specifically defined ROIs. In detail, for dMR images, we averaged the signal intensity within a CC splenial mask in each subject *B*0 space derived from the standard atlas in FSL. The atlas was created by hand segmentation of a standard‐space average of DTI maps from 81 subjects from the ICBM DTI workshop. For the resting‐state fMRI scan, the mask we used was the significantly changed VMHC cluster in each subject's native functional space that had been generated above.

## Results

### Physiological and behavioral findings

Compared with the controls, HA immigrants showed significantly lower values in vital capacity (VC), forced vital capacity (FVC), and forced expiratory volume (FEV) in one second, at 25%, and at 75% (Table 1). HA immigrants showed significantly higher hemoglobin and red blood cell concentrations when compared with SL controls. There were no significant differences between HA immigrants and SL controls in anxiety (HA immigrants = 41.67 ± 9.59, SL controls = 40.33 ± 9.32; *P *=* *0.702) or depression (HA immigrants = 42.69 ± 15.05, SL controls = 38.00 ± 7.53; *P *=* *0.266) scores. The results of the neuropsychological tests are shown in Table 2. Compared with controls, HA immigrants showed increased reaction time on number search, memory search, and mental rotation tasks. Moreover, HA immigrants had lower scores on the standard score of the mental rotation test. There were no significant differences between groups on the digit span, visual reproduction, or ROCF tasks.

**Table 2:** Results of neuropsychological tests in HA immigrants and SL controls

Test	HA immigrants	SL controls	*P*	*d*	Minimum *N*
Memory search					
Standard score	7.0 ± 0.8	6.2 ± 1.5	0.064	0.67	36
Reaction time (ms)	6300.9 ± 430.3	5616.3 ± 425.2	**0.001**	1.60	8
Number search					
Standard score	4.8 ± 1.6	4.1 ± 2.7	0.324	0.32	155
Reaction time (ms)	6730.9 ± 648.3	6225.7 ± 418.5	**0.029**	0.93	20
Mental rotation					
Standard score	1.3 ± 0.9	2.9 ± 2.8	**0.045**	−0.77	28
Reaction time (ms)	15038.9 ± 4181.0	8654.3 ± 684.3	**0.001**	2.13	5
Digit span					
Forward task	8.7 ± 1.2	8.9 ± 1.1	0.756	−0.17	545
Backward task	6.7 ± 1.5	6.5 ± 1.3	0.713	0.14	802
Rey‐Osterrieth Complex Figure					
Immediate recall	26.0 ± 6.3	27.3 ± 6.9	0.642	−0.20	394
Delayed recall	25.8 ± 6.8	28.0 ± 6.4	0.419	−0.33	146
Visual reproduction	13.2 ± 1.8	13.9 ± 0.3	0.145	−0.54	55

The *P*‐values in bold indicate a significance of *P *<* *0.05; Effect size (*d*) was computed according to the formula presented by Cohen. The minimum *N* refers to the equal number of subjects in each group we need to recruit to detect a significant change at the 0.05 level corresponding to a power of 0.8 and the effect sizes (*d*) we reported here.

Unless otherwise indicated, the data are given as the means ± standard deviation.

### Resting‐state fMRI

**Figure 1:**
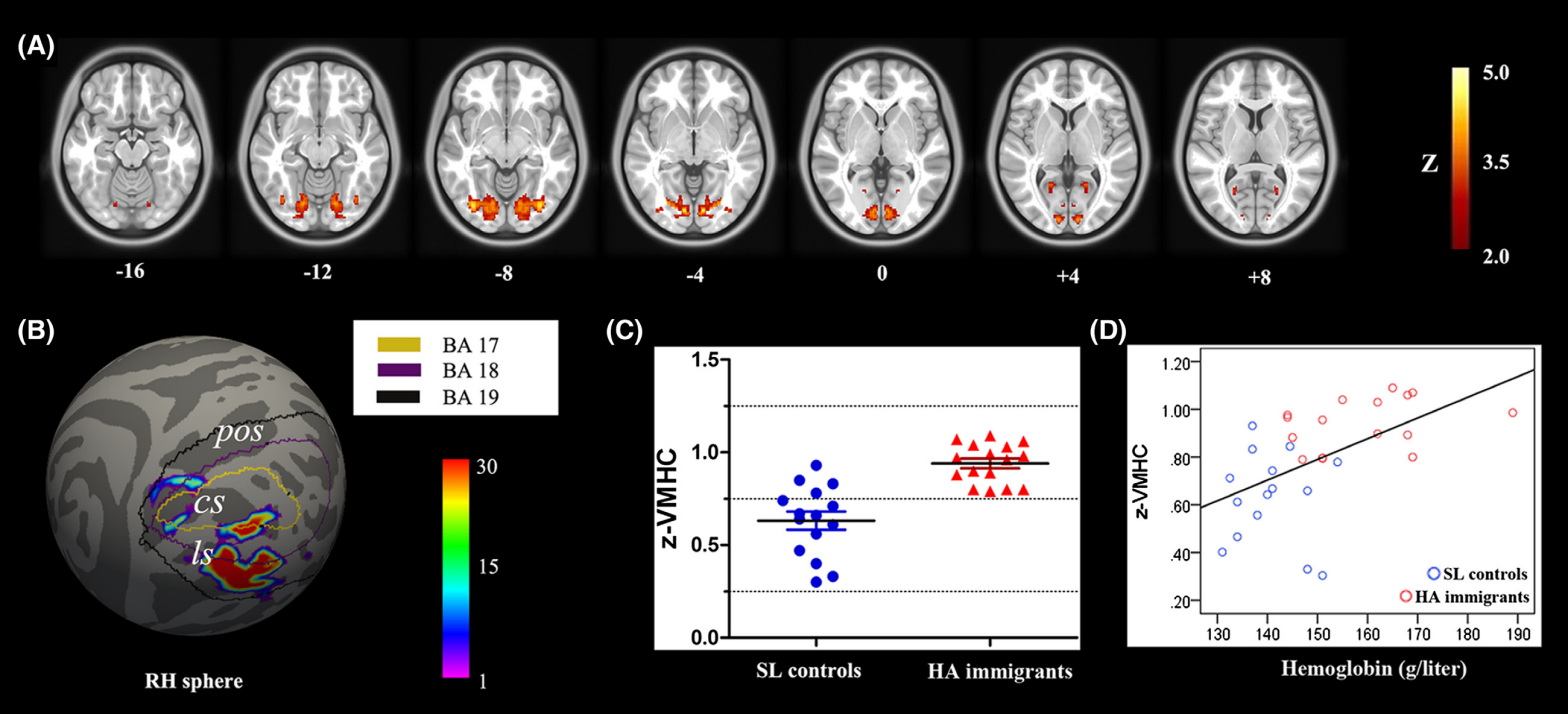
Between group comparison for VMHC. (A) Seven symmetric axial slices show group differences in homotopic voxel‐mirrored connectivity (VMHC). The visual area for which the HA group exhibited significantly stronger VMHC than the control group (Z > 2.57, cluster level *P *<* *0.05, corrected). (B) For a more accurate spatial localization, population‐based probabilistic maps of the significantly increased VMHC regions (visual cortex) were projected onto the FreeSurfer‐derived sphere surface. The outlines were labels according to Brodmann area (BA) 17, 18, and 19. (C). Scatterplots show the between‐group differences in VMHC in the bilateral visual cortex. (D) Correlation between the mean z‐VMHC index and the hemoglobin concentration in both SL controls and HA adults (*P *<* *0.05, Bonferroni corrected). RH, right hemisphere; *cs*, calcarine sulcus; *pos*, parietooccipital sulcus; *ls*, lingual sulcus.

One subject in the control group was excluded for further analysis because of excessive head movement (>2 mm translation). SL controls and HA immigrants differed on global mean VMHC. Specifically, global VMHC was significantly increased in the HA group (HA immigrations = 0.45 ± 0.03; SL controls = 0.40 ± 0.06; *t* = 2.740, *P *=* *0.01). Compared to SL controls, regional differences in VMHC were detected in the bilateral visual cortex with an increase in HA immigrants (*P *<* *0.05, corrected) (Fig. 1; Table 3) controlling for age and years of education. No significant differences were detected in the SL controls >HA immigrants contrast after correction for multiple comparisons. According to Figure 1B, the significant clusters of the visual cortex for each subject were mainly located in the calcarine, parietooccipital, and lingual sulci. The results did not change after controlling for the hematocrit level or voxel gray matter volume in the statistical analysis.

**Table 3:** Regions showing different interhemispheric functional connectivity between two groups

Region	Number of voexls	BA	MNI coordinate			Z^a^
			x	y	z	
Visual cortex	1504	17/18/19	±8	−84	−2	4.015

BA, Brodmann area.

a Maximum z‐statistic.

Averaged z‐VMHC extracted from bilateral visual cortex showed a significantly positive correlation with hemoglobin concentration after Bonferroni correction (*r *=* *0.545, *P *=* *0.002) (Fig. 1D). We did not find a significant correlation between the z‐VMHC within the visual cortex and other physiological and behavior measures (all *P*‐values >0.05). Whole‐brain correlation analysis found no significant correlations between z‐VMHC and altitude, quadratic altitude or cubic altitude after GRF correction. Adding age and/or education as a covariate did not alter the correlation findings for cognitive measurements.

### DTI

**Figure 2:**
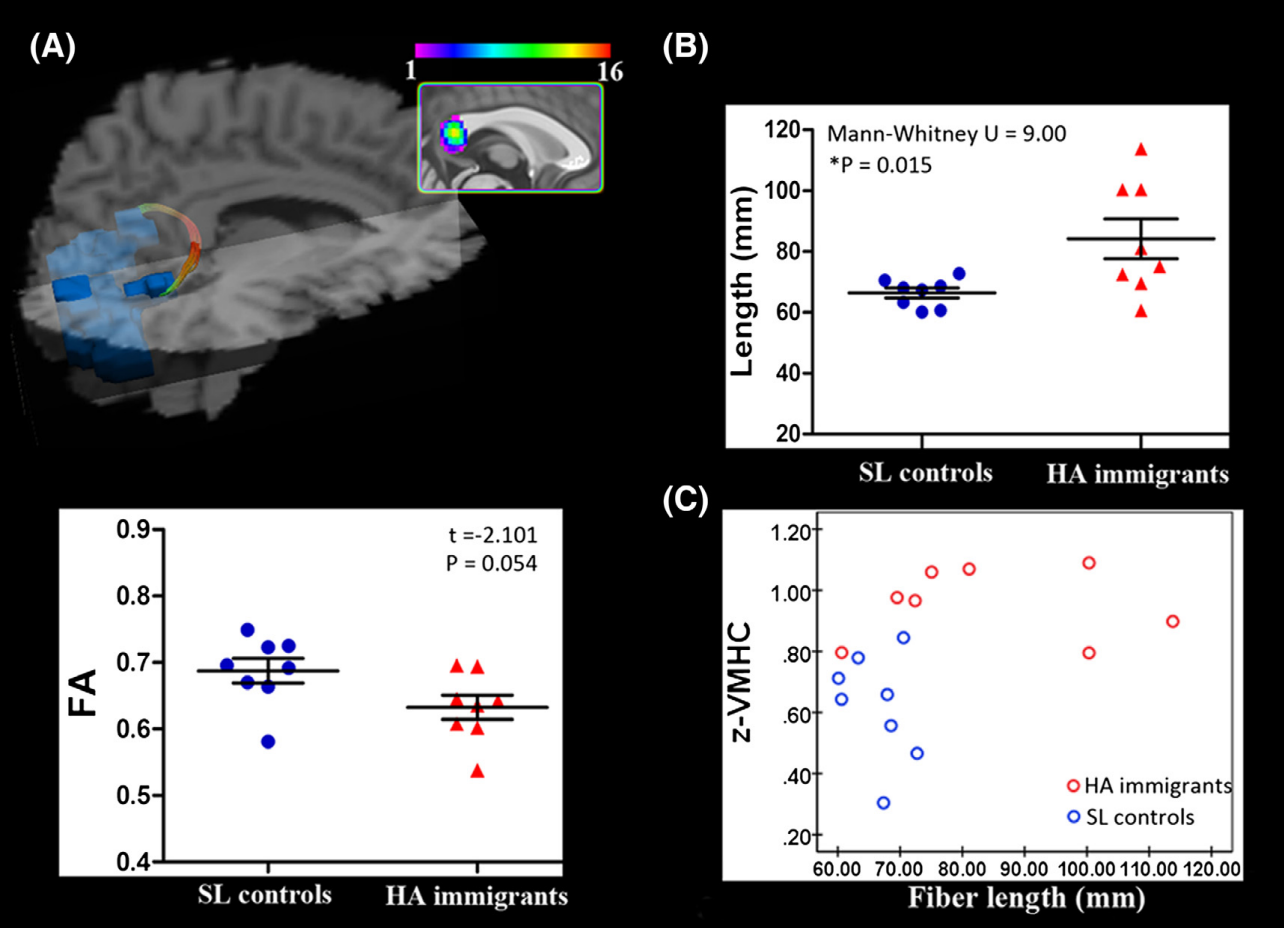
Diffusion tractographic images from a single control subject show between‐group comparison for commissural fiber parameters. (A) Diffusion tractographic images from a single control subject. Fibers connecting the bilateral visual cortex are located in the splenium of the corpus callosum. The inset shows the probabilistic maps of the commissural tract constructed with data from 16 subjects. (B) Scatterplots show the between‐group comparison for the commissural fiber parameters of fractional anisotropy (FA) and fiber length. *P <* *0.05, Bonferroni corrected. (C) A significant correlation between mean z‐VMHC index within homotopic visual areas and the length of their commissural fibers. Spearman rho = 0.565, *P *=* *0.023. A longer path length of the fibers connecting the bilateral visual cortex corresponds to stronger interhemispheric functional synchronization. SL, sea level; HA, high‐altitude.

The commissural tracts connecting the bilateral visual cortex was not detected for eight of the 15 subjects in the SL group and eight of the 16 subjects in the HA group. Figure 2A shows a diffusion tractographic image from a single control subject. The results found here were in line with previous studies that interindividual differences exist in splenial connectivity, and homotopic connections between the primary visual cortices are variable (Putnam et al. 2010). The distribution of the individuals with detected tracts were comparable between the two groups (Chi‐square = 0.034, *P *=* *1). Therefore, the following structural‐functional correlations were calculated only for the subjects with reconstructed fiber tracts. The between‐group analysis indicated that the reconstructed commissural fibers connecting the bilateral visual cortex were significantly longer (Mann–Whitney *U* = 9.00, *P *=* *0.015) and the FA was smaller and approached significance (*t* = −2.101, *P *=* *0.054) in HA immigrants (Fig. 2B). To clearly show through which part of the corpus callosum these fibers went, we produced a population‐based (*n* = 16) probabilistic map. We found that the fibers connecting the bilateral visual cortex were located in the splenium of the corpus callosum. Using Pearson correlations, fiber parameters (fiber length, FA) were not significantly correlated with neuropsychological measurements, physiological indices, or altitude (all *P*‐values >0.05).

### Relationship between functional and structural connectivity

Fiber length did not conform to the normal distribution in the combined group of those HA immigrants and controls with reconstructed tracts (individuals with reconstructed tracts both in HA and SL groups) (*P *=* *0.006, one‐sample Kolmogorov–Smirnov test), and spearman rho correlation analysis was thus adopted. A significant positive correlation between mean z‐VMHC of the bilateral visual cortex and the length of reconstructed commissural fibers was detected (rho = 0.565, *P *=* *0.023, corrected) (Fig. 2C). No significant correlation was found between FA and z‐VMHC (*P *=* *0.253).

For resting‐state data, the effect size (*d*) for global VMHC was 1.05, and the effect size for altered regional VMHC within the visual cortex was 1.97. Two fiber parameters of FA and fiber length were involved. The effect size for FA was 1.12 and for fiber length was 1.32. All these reported effect sizes are large (Cohen 1977). The minimum subject numbers (*N*) for these behavioral, physiological, resting‐state, and DTI data were given according to the effect sizes shown above and were also based on the significant level *α* of 0.05 and a statistical power of 0.8. Specifically, the minimum required *N* for global VMHC was 16, for regional VMHC within the visual cortex was 6, for FA was 14, and for fiber length was 11. The detailed values for behavior and physiological data are all shown in Tables 1 and 2.

### Additional SNR analysis

**Figure 3:**
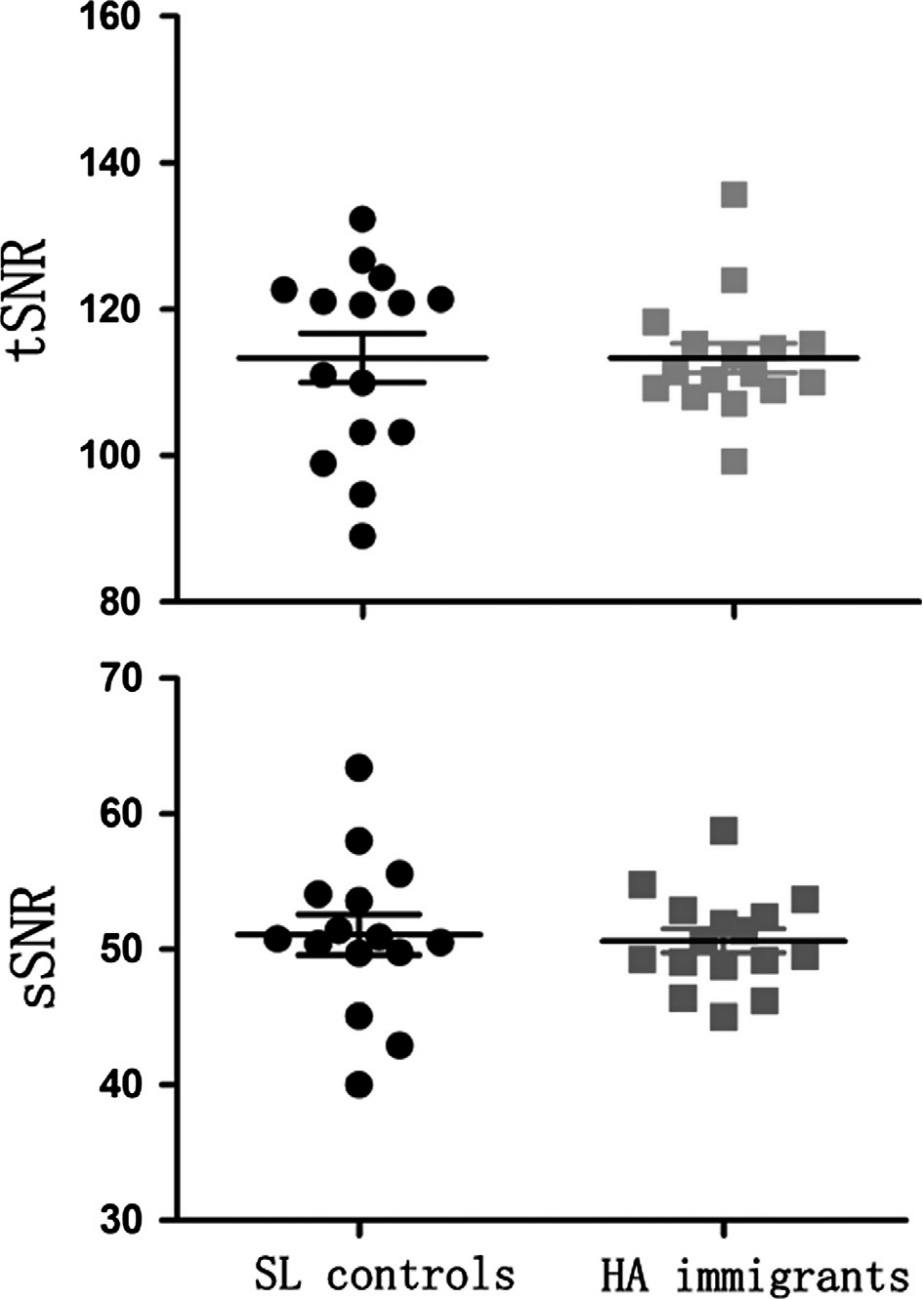
Scatterplots show the between‐group comparisons for the time‐series signal‐to‐noise ratio (tSNR) and spatial SNR (sSNR). SL, sea level; HA, high‐altitude.

Neither the sSNR nor the tSNR showed significant changes in HA immigrants when compared with SL controls (tSNR: HA immigrations = 113.30 ± 8.07, SL controls = 113.33 ± 12.95; *t* = 0.008, *P *=* *0.994; sSNR: HA immigrations = 50.63 ± 3.50, SL controls = 51.08 ± 5.77; *t* = 0.267, *P *=* *0.791) (Fig. 3).

## Discussion

In this study, we investigated the compensatory modifications of resting‐state functional architecture (VMHC) in the adult brain from long‐term adaptation to chronic HA hypoxia, and also investigated the anatomical connectivity (fiber length and FA derived from DTI tractography) that underlies the changed homotopic functional connectivity.

### Interhemispheric functional connectivity

Functional homotopy reflects an essential aspect of brain function (Salvador et al. 2008). In this study, significantly increased interhemispheric functional connectivity was found in the visual cortex, which encompassed the three cytoarchitectonic distinct Brodmann areas 17, 18, and 19 (Fig. 1). Phototransduction and visual input transmission are primarily affected in HA immigrants (Schatz et al. 2013). Furthermore, cytoarchitectonic area 17 (visual field 1 or area striata) receives visual inputs from the thalamic relay of the optic pathway (i.e., the lateral geniculate complex), which is one of the most damaged structures in hypoxic conditions (Huang and Castillo 2008). These low‐level deficits appear to upwardly generalize to other high‐order cognitive deficits observed in HA exposure adults, such as long‐term visual memory, visual discrimination, visual attention, and visual learning (Virués‐Ortega et al. 2004). The hypoxic environment disturbs the visual conducting pathway, and feedforward regulation could change the cortical equilibrium and lead to functional reorganization. Homotopic connectivity is an important aspect of interhemispheric communication for bilateral hemispheres to integrate brain function underlying coherent cognition and behavior, which is particularly required for visual function because it is indeed based on the fusion of unilateral visual objects into continuous percepts (Berlucchi 2014). For some hemispheric dominant visual functions, such as visuospatial attention, more neurons may be triggered and fire synchronously to the contralateral half of the visual cortex to compensate for functional deficits in the unilateral visual cortex. Supporting this conclusion, results from a recent event‐related potential study in HA immigrants indicated that hemispheric compensation exists in the visual cortex during discrimination processes at early visuospatial attention processing stages (Wang et al. 2014). The enhancement of functional synchronization in the adult brain may serve as an underlying mechanism to compensate for neuronal cost and to maintain cognitive demands living and working in an HA environment. Moreover, averaged VMHC within the significantly changed visual cortex was positively correlated with hemoglobin concentration (Fig. 1C), which indicates that brain functional modification, to some extent, is related to the status of physiological adaptation.

As our previous brain morphological study in this same sample found no significant GM difference in the visual cortex between the HA and control groups (Zhang et al. 2013), the significantly increased VMHC in HA immigrants should not arise from the structural differences between the two groups.

### Anatomical connectivity of the increased VMHC regions

Two basic indices of the reconstructed commissural fibers were measured, including path length and FA. FA is regarded as an indicator of the diameter and density of fibers, myelination, and macrostructural features (such as fiber tract coherence) of WM fibers (Basser and Pierpaoli 2011), which we found here approached a significant decrease in HA immigrants. This result was in compliance with our previous whole‐brain tract‐based spatial statistical study that FA in the forceps major of the CC was decreased (Zhang et al. 2013). Compared to the damage in the axonal membrane, the path length of the commissural fibers connecting the bilateral visual cortex was increased in HA immigrants, correlating positively with the increased z‐VMHC. Tract length has been used to examine WM integrity and connectivity efficiently in a number of healthy and diseased brains (Upadhyay et al. 2007; Ji et al. 2014). A long‐distance connection may be important for complex neural systems because they are not exclusively optimized for minimal global wiring length, but rather for a variety of factors including the minimization of processing steps (Kaiser and Hilgetag 2006). Mapping and assessing how short or long fibers are topographically distributed can extend our understanding of the underpinning of long‐range information transmission. For midline fusion structures, such as the visual cortex, the long‐distance callosal connections are by far the most important pathway for each hemisphere to receive visual information from the ipsilateral half of the visual field. In this study, the modified fibers may optimize the connectivity efficiency to compensate for the compromised medullary structure.

The visual cortex and its connecting fibers were demonstrated to be associated with changes in environmental demands (Zatorre et al. 2012) and the plastic capacity retained throughout life (Gilbert and Li 2012). Although there have been rapid progress in imaging methods and computational algorithms, it is still difficult for MRI measures to interpret the cellular and molecular events underlying the changed anatomical connectivity. The potential explanations may be axonal sprouting, pruning, or rerouting on the basis of previous literature (Zatorre et al. 2012). With respect to the visual cortex, fiber rewiring has been observed in response to brain injury in a squirrel monkey model (Dancause et al. 2005), and similar rebranching could also have been induced to allow particular types of visual information to reach parietal regions after training (Johansen‐Berg 2007). Furthermore, previous studies have confirmed that fiber connections between bilateral visual fields undergo rapid and exuberant sprouting and pruning to facilitate topographic reorganization in recovery from central nerve system damage (Gilbert and Li 2012), and specific neurons in the adult brain also carry a latent neurogenic program even after hypoxic exposure (Zhu et al. 2005). In the setting of an adult brain in adaptation to long lasting inspiratory hypoxia, which of the above mentioned events triggers or dominates the DTI‐derived fiber changes needs to be further identified. Changes in the route of fiber bundles should affect imaging measures reflecting the directional preferences of water diffusion. DTI tractography relies on predefined FA values and the combination of rotation angle directions, and thus is sensitive to detecting subtle changes in tract geometry. The reinforced direct monosynaptic connections between homotopic visual areas are likely to maintain normal vision and to compensate for the compromised visual functions.

In this study, HA immigrants showed increased reaction times during number search, memory search and mental rotation tasks, which were in line with the previous literature (Virués‐Ortega et al. 2004). Increases in reaction times have also been found in HA immigrant descendants during performance of spatial and verbal working memory tasks (Yan et al. 2011a,b). On the other hand, previous neuropsychological studies in mountain climbers, healthy males with exposure to a simulated altitude of 4500 m for 2 days, showed poorer performance on digit span, visual reproduction, and ROCF tasks (Virués‐Ortega et al. 2004; Aquino Lemos et al. 2012). However, in this study, these behaviors in HA immigrants were comparable with the SL controls, which may indicate restorative processes in brain function and structure to compensate for the cognitive deficits after years of adaptation. However, we did not find any relationships between brain parameters and cognitive tests.

### Relationship between interhemispheric functional and structural connectivity

Structural connectivity provides the material substrate for functional connectivity (van den Heuvel et al. 2009; Honey et al. 2009), the architecture of which could in turn influence the transmission of neural activity (Schulte et al. 2005). Here, we further performed tractographic analysis to clarify whether the increased interhemispheric functional connectivity was associated with corresponding alterations of anatomical connectivity. The results showed that both the functional and structural connectivity were reinforced in HA immigrants when compared with SL controls. Furthermore, the length of the fibers connecting homotopic visual areas correlated positively with the averaged VMHC value. According to previous studies, modifications of these two connective modalities could affect interaction (Honey et al. 2009; Tovar‐Moll et al. 2014), and it is widely assumed that structural connectivity is relatively stable over time, whereas functional connectivity is more variable (Zhang et al. 2011). Therefore, we hypothesized that homotopic functional brain reorganization was initially triggered to compensate for the compromised microstructural integrity of transcallosal WM fibers, and the functional deficits in the unilateral visual cortex, such as long‐term interhemispheric functional enhancement, may induce a dynamic modification of structural connectivity. Finally, the modified structural connectivity could reinforce the synchrony of neuron firing and contribute to the maintenance of a stable enhancement of homotopic functional connectivity. The presentation of both anatomical and functional modifications suggested that the visual cortex is critical for the adult brain in adaptation to an HA environment. Future studies, however, will be needed to clarify the underlying mechanism of the changed functional and structural connectivity and their causal relationships, which at present are speculative.

### Hemodynamic considerations

The BOLD signal is related to vascular effects (Liu 2013). During HA exposure, the immigrants should experience multiple hemodynamic changes, including increases in red blood cell and hemoglobin concentrations, a reduction in arterial blood oxygen saturation and higher binding affinity of hemoglobin to oxygen (Zubieta‐Calleja et al. 2007; Wilson et al. 2009). These changed hemodynamic events should alter the T2/T2* value of the brain tissue, and altered the T2/T2* that causes SNR change in fMRI and dMRI (Uematsu et al. 2007), which may in turn affect the VMHC and tractographic measures. Here, in addition to performing FIX to rule out nuisance signals (Murphy et al. 2013; Salimi‐Khorshidi et al. 2014), we further performed an analysis for SNRs within the bilateral visual cortex and the CC to validate our results. We did not find any significant differences (either in sSNR or tSNR) between the HA and SL groups, which suggested that group differences in the VMHC and tractographic measurements were not likely from the supposed SNR changes due to variations in hemoglobin content and hemodynamic events.

One recent study concerned the impact of hematocrit level on resting‐state functional brain connectivity in healthy subjects revealed a positive correlation between the hematocrit level and VMHC measure only in the supramarginal gyrus (Yang et al. 2014). In this study, we observed that hemoglobin concentration was positively correlated with the value of VMHC within the visual cortex. This divergence could be largely attributed to the fact that HA immigrants have many more neural and hemodynamic adaptation processes than that in healthy individuals. Moreover, the relationship between structural and functional connectivity could further confirm the neural effect in contribution to the resulting metrics because the changed structural connectivity would modulate the transmission of neural discharge and affect the neural activity synchronization. Although the blood physiology will systematically impact the BOLD time series, the hemodynamic uncertainty is ubiquitously accompanied with HA immigration and thus is an interesting insight into the neural and vascular relationship with respect to functional brain MR imaging. Future studies that add a noise in the images may be of value to estimate how degrading the SNR may affect the results of VMHC and diffusion‐based tractography, which will help us to delineate neuronal activities or non‐neuronal noises in the studies involving populations who have physiologically or pathologically baseline hemodynamic changes.

### Limitations

Several limitations of this study should be recognized. First, this is a small sample study that has nonetheless provided valuable information for preparing our follow‐up with larger samples and longitudinal designs. Living elsewhere may be influenced by other factors (e.g., differences in climate or culture). These factors may bring about differentially emotional effects in each individual, leading to within‐group variation in brain function that is not of interest. To reduce such potential false positives in the group‐level analysis, we adopted a relatively conservative multiple comparison correction method, GRF (two‐tailed Z > 2.57, cluster level *P *<* *0.05). Furthermore, the HA immigrants did not show significant differences in anxiety and depression scores when compared with SL controls. Second, regarding the VMHC method, although the primary limitation may be attributed to the fact that the brain is not symmetrical, we used the averaged and mirrored structural images as our study with a specific symmetrical standard template, and also performed spatial smoothing to improve the functional correspondence between homotopic voxels. Third, even though DTI has been commonly used to analyze WM connectivity, it has inherent limitations. The limited spatial resolution of conventional DTI and its applications with a three Tesla scanner show low sensitivity such that a large number of connecting fiber tracts were undetected, especially in regions of complex fiber geometry (e.g., crossing fibers). According to the fiber tracing algorithm, we employed the method of deterministic tractography that could effectively avoid invalid fibers in the reconstruction step (Côté et al. 2013). Lastly, significant correlations between cognitive measurements and functional and fiber parameters were not detected. Examining the longitudinal change in cognition in HA immigrants would be a relatively more robust method of exploring relationships between brain functional and structural compensatory mechanisms and the restorative processes of cognition and behavior.

## Conclusion

In this study, we fused resting‐state functional connectivity and DTI tractography techniques to reveal a new mechanistic avenue in the brain of HA immigrants to compensate for neurologic deficits independent of inherited and developmental effects. We demonstrated, for the first time, that VMHC in the visual cortex was significantly enhanced, and the length of WM fibers connecting homotopic visual areas was significantly increased in HA immigrants when compared with SL controls. Furthermore, VMHC in the bilateral visual cortex was positively correlated with hemoglobin concentration and the measured length of the commissural fibers. These observations are the first to demonstrate adult brain functional and structural connectivity resilience after long‐term HA exposure, extending the previously proposed brain hypometabolic mechanism, and the coupled modifications among the bilateral visual cortex indicate important neural compensatory mechanisms underlying visual dysfunction in HA immigrants. Successful physiological adaptation may constitute the basis for brain functional reorganization and dynamic structural remodeling, especially for an increase in hemoglobin concentration, which is pivotal for brain oxygen consumption and cellular energy supply. Studies of human central adaptation to extreme environments promote the understanding of our brain's capacity for survival.

## Conflict of Interest

The authors declare no conflict of interest.

## References

[brb3512-bib-0001] Anderson, J. S. , T. J. Druzgal , A. Froehlich , M. B. DuBray , N. Lange , A. L. Alexander , et al. 2010 Decreased interhemispheric functional connectivity in autism. Cereb. Cortex 21:1134–1146. 20943668 10.1093/cercor/bhq190 PMC3077433

[brb3512-bib-0002] Aquino Lemos, V. , H. K. M. Antunes , R. V. T. Santos , F. S. Lira , S. Tufik , and M. T. Mello . 2012 High altitude exposure impairs sleep patterns, mood, and cognitive functions. Psychophysiology 49:1298–1306. 22803634 10.1111/j.1469-8986.2012.01411.x

[brb3512-bib-0003] Basser, P. J. , and C. Pierpaoli . 2011 Microstructural and physiological features of tissues elucidated by quantitative‐diffusion‐tensor MRI. J. Magn. Reson. 213:560–570. 22152371 10.1016/j.jmr.2011.09.022

[brb3512-bib-0004] Beall, E. B. , and M. J. Lowe . 2007 Isolating physiologic noise sources with independently determined spatial measures. NeuroImage 37:1286–1300. 17689982 10.1016/j.neuroimage.2007.07.004

[brb3512-bib-0005] Beckmann, C. F. , and S. M. Smith . 2004 Probabilistic independent component analysis for functional magnetic resonance imaging. IEEE Trans. Med. Imaging 23:137–152. 14964560 10.1109/TMI.2003.822821

[brb3512-bib-0006] Berlucchi, G. 2014 Visual interhemispheric communication and callosal connections of the occipital lobes. Cortex 56:1–13. 23489777 10.1016/j.cortex.2013.02.001

[brb3512-bib-0007] Chen, J. , I.‐T. Lin , H. Zhang , J. Lin , S. Zheng , M. Fan , et al. 2015 Reduced cortical thickness, surface area in patients with chronic obstructive pulmonary disease: a surface‐based morphometry and neuropsychological study. Brain Imaging Behav. doi:10.1007/s11682-015-9403-7 [Epub ahead of print]. 10.1007/s11682-015-9403-7 25986304

[brb3512-bib-0008] Chen, J. , C. Fan , J. Li , Q. Han , J. Lin , T. Yang , et al. 2016 Increased intraregional, synchronized neural activity in adult brain after prolonged adaptation to high‐altitude hypoxia: a resting‐state fMRI study. High Alt. Med. Biol. 17:16–24. 26906285 10.1089/ham.2015.0104

[brb3512-bib-0009] Cohen, J. 1977 Statistical power analysis for the behavioral sciences. Academic Press, New York.

[brb3512-bib-0010] Côté, M. A. , G. Girard , A. Boré , E. Garyfallidis , J. C. Houde , and M. Descoteaux . 2013 Tractometer: towards validation of tractography pipelines. Med. Image Anal. 17:844–857. 23706753 10.1016/j.media.2013.03.009

[brb3512-bib-0011] Dancause, N. , S. Barbay , S. B. Frost , E. J. Plautz , D. Chen , E. V. Zoubina , et al. 2005 Extensive cortical rewiring after brain injury. J. Neurosci. 25:10167–10179. 16267224 10.1523/JNEUROSCI.3256-05.2005 PMC6725801

[brb3512-bib-0013] Fox, M. D. , A. Z. Snyder , J. L. Vincent , M. Corbetta , D. C. Van Essen , and M. E. Raichle . 2005 The human brain is intrinsically organized into dynamic, anticorrelated functional networks. Proc. Natl Acad. Sci. USA 102:9673–9678. 15976020 10.1073/pnas.0504136102 PMC1157105

[brb3512-bib-0014] Friedman, L. , and G. H. Glover . 2006 Report on a multicenter fMRI quality assurance protocol. J. Magn. Reson. Imaging 23:827–839. 16649196 10.1002/jmri.20583

[brb3512-bib-0015] Gilbert, C. D. , and W. Li . 2012 Adult visual cortical plasticity. Neuron 75:250–264. 22841310 10.1016/j.neuron.2012.06.030 PMC3408614

[brb3512-bib-0016] Gong, P. , A. Zheng , D. Chen , W. Ge , C. Lv , K. Zhang , et al. 2009 Effect of BDNF Val66Met polymorphism on digital working memory and spatial localization in a healthy Chinese Han population. J. Mol. Neurosci. 38:250–256. 19424874 10.1007/s12031-009-9205-8

[brb3512-bib-0018] Griffanti, L. , G. Salimi‐Khorshidi , C. F. Beckmann , E. J. Auerbach , G. Douaud , C. E. Sexton , et al. 2014 ICA‐based artefact removal and accelerated fMRI acquisition for improved resting state network imaging. NeuroImage 95:232–247. 24657355 10.1016/j.neuroimage.2014.03.034 PMC4154346

[brb3512-bib-0019] van den Heuvel, M. P. , R. C. Mandl , R. S. Kahn , H. Pol , and E. Hilleke . 2009 Functionally linked resting‐state networks reflect the underlying structural connectivity architecture of the human brain. Hum. Brain Mapp. 30:3127–3141. 19235882 10.1002/hbm.20737 PMC6870902

[brb3512-bib-0020] Hochachka, P. , C. Clark , W. Brown , C. Stanley , C. Stone , R. Nickles , et al. 1994 The brain at high altitude: hypometabolism as a defense against chronic hypoxia? J. Cereb. Blood Flow Metab. 14:671–679. 8014215 10.1038/jcbfm.1994.84

[brb3512-bib-0021] Hochachka, P. , L. Buck , C. Doll , and S. Land . 1996 Unifying theory of hypoxia tolerance: molecular/metabolic defense and rescue mechanisms for surviving oxygen lack. Proc. Natl Acad. Sci. USA 93:9493–9498. 8790358 10.1073/pnas.93.18.9493 PMC38456

[brb3512-bib-0022] Honey, C. , O. Sporns , L. Cammoun , X. Gigandet , J.‐P. Thiran , R. Meuli , et al. 2009 Predicting human resting‐state functional connectivity from structural connectivity. Proc. Natl Acad. Sci. USA 106:2035–2040. 19188601 10.1073/pnas.0811168106 PMC2634800

[brb3512-bib-0023] Hu, W. D. , T. Wang , X. J. Li , and D. M. Miao . 1999 Development of group psychological measurement multimedia system. J. Fourth Milit. Med. Univ. 3:225–227.

[brb3512-bib-0024] Huang, B. Y. , and M. Castillo . 2008 Hypoxic‐ischemic brain injury: imaging findings from birth to adulthood 1. Radiographics 28:417–439. 18349449 10.1148/rg.282075066

[brb3512-bib-0025] Hunt, J. S. , R. J. Theilmann , Z. M. Smith , M. Scadeng , and D. J. Dubowitz . 2013 Cerebral diffusion and T2: MRI predictors of acute mountain sickness during sustained high‐altitude hypoxia. J. Cereb. Blood Flow Metab. 33:372–380. 23211961 10.1038/jcbfm.2012.184 PMC3587813

[brb3512-bib-0026] Innocenti, G. M. 1986 General organization of callosal connections in the cerebral cortex Pp. 291–353 *in* Jones E. G., and Peters A., eds. Sensory‐motor areas and aspects of cortical connectivity. Springer, New York: Plenum.

[brb3512-bib-0027] Jenkinson, M. , P. Bannister , M. Brady , and S. Smith . 2002 Improved optimization for the robust and accurate linear registration and motion correction of brain images. NeuroImage 17:825–841. 12377157 10.1016/s1053-8119(02)91132-8

[brb3512-bib-0028] Ji, G.‐J. , Z. Zhang , Q. Xu , Y.‐F. Zang , W. Liao , and G. Lu . 2014 Generalized tonic‐clonic seizures: aberrant interhemispheric functional and anatomical connectivity. Radiology 271:839–847. 24588676 10.1148/radiol.13131638

[brb3512-bib-0029] Johansen‐Berg, H. 2007 Structural plasticity: rewiring the brain. Curr. Biol. 17:R141–R144. 17307051 10.1016/j.cub.2006.12.022

[brb3512-bib-0030] Kaiser, M. , and C. C. Hilgetag . 2006 Nonoptimal component placement, but short processing paths, due to longdistance projections in neural systems. PLoS Comput. Biol. 2:e95. 16848638 10.1371/journal.pcbi.0020095 PMC1513269

[brb3512-bib-0031] Leemans, A. , and D. K. Jones . 2009 The B‐matrix must be rotated when correcting for subject motion in DTI data. Magn. Reson. Med. 61:1336–1349. 19319973 10.1002/mrm.21890

[brb3512-bib-0032] Liu, T. T. 2013 Neurovascular factors in resting‐state functional MRI. NeuroImage 80:339–348. 23644003 10.1016/j.neuroimage.2013.04.071 PMC3746765

[brb3512-bib-0033] Lowe, M. J. , B. J. Mock , and J. A. Sorenson . 1998 Functional connectivity in single and multislice echoplanar imaging using resting‐state fluctuations. NeuroImage 7:119–132. 9558644 10.1006/nimg.1997.0315

[brb3512-bib-0035] Muotri, A. R. , and F. H. Gage . 2006 Generation of neuronal variability and complexity. Nature 441:1087–1093. 16810244 10.1038/nature04959

[brb3512-bib-0100] Murphy, K. , R. M. Birn , and P. A. Bandettini . 2013 Resting‐state fMRI confounds and cleanup. Neuroimage 80:349–359. 23571418 10.1016/j.neuroimage.2013.04.001 PMC3720818

[brb3512-bib-0036] Parrish, T. B. , D. R. Gitelman , K. S. LaBar , and M. Mesulam . 2000 Impact of signal‐to‐noise on functional MRI. Magn. Reson. Med. 44:925–932. 11108630 10.1002/1522-2594(200012)44:6<925::aid-mrm14>3.0.co;2-m

[brb3512-bib-0037] Pascual‐Leone, A. , A. Amedi , F. Fregni , and L. B. Merabet . 2005 The plastic human brain cortex. Annu. Rev. Neurosci. 28:377–401. 16022601 10.1146/annurev.neuro.27.070203.144216

[brb3512-bib-0038] Putnam, M. C. , M. S. Steven , K. W. Doron , A. C. Riggall , and M. S. Gazzaniga . 2010 Cortical projection topography of the human splenium: hemispheric asymmetry and individual differences. J. Cogn. Neurosci. 22:1662–1669. 19583478 10.1162/jocn.2009.21290

[brb3512-bib-0039] Richardson, C. , A. M. Hogan , R. S. Bucks , A. Baya , J. Virues‐Ortega , J. W. Holloway , et al. 2011 Neurophysiological evidence for cognitive and brain functional adaptation in adolescents living at high altitude. Clin. Neurophysiol. 122:1726–1734. 21377415 10.1016/j.clinph.2011.02.001

[brb3512-bib-0041] Salimi‐Khorshidi, G. , G. Douaud , C. F. Beckmann , M. F. Glasser , L. Griffanti , and S. M. Smith . 2014 Automatic denoising of functional MRI data: combining independent component analysis and hierarchical fusion of classifiers. NeuroImage 90:449–468. 24389422 10.1016/j.neuroimage.2013.11.046 PMC4019210

[brb3512-bib-0042] Salvador, R. , A. Martinez , E. Pomarol‐Clotet , J. Gomar , F. Vila , S. Sarro , et al. 2008 A simple view of the brain through a frequency‐specific functional connectivity measure. NeuroImage 39:279–289. 17919927 10.1016/j.neuroimage.2007.08.018

[brb3512-bib-0043] Schatz, A. , G. Willmann , M. D. Fischer , K. Schommer , A. Messias , E. Zrenner , et al. 2013 Electroretinographic assessment of retinal function at high altitude. J. Appl. Physiol. 115:365–372. 23722709 10.1152/japplphysiol.00245.2013

[brb3512-bib-0044] Schulte, T. , E. V. Sullivan , E. Müller‐Oehring , E. Adalsteinsson , and A. Pfefferbaum . 2005 Corpus callosal microstructural integrity influences interhemispheric processing: a diffusion tensor imaging study. Cereb. Cortex 15:1384–1392. 15635059 10.1093/cercor/bhi020

[brb3512-bib-0045] Shmueli, K. , P. van Gelderen , J. A. de Zwart , S. G. Horovitz , M. Fukunaga , J. M. Jansma , et al. 2007 Low‐frequency fluctuations in the cardiac rate as a source of variance in the resting‐state fMRI BOLD signal. NeuroImage 38:306–320. 17869543 10.1016/j.neuroimage.2007.07.037 PMC2128785

[brb3512-bib-0046] Song, M. , J.‐H. Chen , J. Chen , and I.‐T. Lin . 2015 Comparisons between the 35 mm quadrature surface resonator at 300 K and the 40 mm high‐temperature superconducting surface resonator at 77 K in a 3T MRI imager. PLoS One 10:e0118892. 25812124 10.1371/journal.pone.0118892 PMC4374922

[brb3512-bib-0047] Stark, D. E. , D. S. Margulies , Z. E. Shehzad , P. Reiss , A. C. Kelly , L. Q. Uddin , et al. 2008 Regional variation in interhemispheric coordination of intrinsic hemodynamic fluctuations. J. Neurosci. 28:13754–13764. 19091966 10.1523/JNEUROSCI.4544-08.2008 PMC4113425

[brb3512-bib-0048] Thomas, C. , K. Humphreys , K.‐J. Jung , N. Minshew , and M. Behrmann . 2011 The anatomy of the callosal and visual‐association pathways in high‐functioning autism: a DTI tractography study. Cortex 47:863–873. 20832784 10.1016/j.cortex.2010.07.006 PMC3020270

[brb3512-bib-0049] Tovar‐Moll, F. , M. Monteiro , J. Andrade , I. E. Bramati , R. Vianna‐Barbosa , T. Marins , et al. 2014 Structural and functional brain rewiring clarifies preserved interhemispheric transfer in humans born without the corpus callosum. Proc. Natl Acad. Sci. USA 111:7843–7848. 24821757 10.1073/pnas.1400806111 PMC4040546

[brb3512-bib-0050] Uematsu, H. , M. Takahashi , H. Hatabu , C. Cl , S. L. Wehrli , F. W. Wehrli , et al. 2007 Changes in T1 and T2 observed in brain magnetic resonance imaging with delivery of high concentrations of oxygen. J. Comput. Assist. Tomogr. 31:662–665. 17895773 10.1097/rct.0b013e3180319114

[brb3512-bib-0051] Upadhyay, J. , M. Ducros , T. A. Knaus , K. A. Lindgren , A. Silver , H. Tager‐Flusberg , et al. 2007 Function and connectivity in human primary auditory cortex: a combined fMRI and DTI study at 3 Tesla. Cereb. Cortex 17:2420–2432. 17190967 10.1093/cercor/bhl150

[brb3512-bib-0052] Van Essen, D. C. 2005 A population‐average, landmark‐and surface‐based (PALS) atlas of human cerebral cortex. NeuroImage 28:635–662. 16172003 10.1016/j.neuroimage.2005.06.058

[brb3512-bib-0053] Virués‐Ortega, J. , G. Buela‐Casal , E. Garrido , and B. Alcázar . 2004 Neuropsychological functioning associated with high‐altitude exposure. Neuropsychol. Rev. 14:197–224. 15796116 10.1007/s11065-004-8159-4

[brb3512-bib-0054] Wang, Y. , H. Ma , S. Fu , S. Guo , X. Yang , P. Luo , et al. 2014 Long‐term exposure to high altitude affects voluntary spatial attention at early and late processing stages. Sci. Rep. 4:4443 http://dx.doi.org/10.1038/srep04443.

[brb3512-bib-0055] Wei, X. , M. Danmin , G. Jingjing , and W. Shengjun . 2007 The construction of the number searching test for nation‐wide conscription. Psychol. Sci. 30:139–141.

[brb3512-bib-0056] Wilson, M. H. , S. Newman , and C. H. Imray . 2009 The cerebral effects of ascent to high altitudes. Lancet Neurol. 8:175–191. 19161909 10.1016/S1474-4422(09)70014-6

[brb3512-bib-0057] Yan, X. , J. Zhang , J. Shi , Q. Gong , and X. Weng . 2010 Cerebral and functional adaptation with chronic hypoxia exposure: a multi‐modal MRI study. Brain Res. 1348:21–29. 20599837 10.1016/j.brainres.2010.06.024

[brb3512-bib-0058] Yan, X. , J. Zhang , Q. Gong , and X. Weng . 2011a Adaptive influence of long term high altitude residence on spatial working memory: an fMRI study. Brain Cogn. 77:53–59. 21767899 10.1016/j.bandc.2011.06.002

[brb3512-bib-0059] Yan, X. , J. Zhang , Q. Gong , and X. Weng . 2011b Prolonged high‐altitude residence impacts verbal working memory: an fMRI study. Exp. Brain Res. 208:437–445. 21107542 10.1007/s00221-010-2494-x

[brb3512-bib-0060] Yang, Z. , R. C. Craddock , and M. P. Milham . 2014 Impact of hematocrit on measurements of the intrinsic brain. Front. Neurosci. 8:452. 25653582 10.3389/fnins.2014.00452 PMC4299407

[brb3512-bib-0061] Zatorre, R. J. , R. D. Fields , and H. Johansen‐Berg . 2012 Plasticity in gray and white: neuroimaging changes in brain structure during learning. Nat. Neurosci. 15:528–536. 22426254 10.1038/nn.3045 PMC3660656

[brb3512-bib-0062] Zhang, J. , X. Yan , J. Shi , Q. Gong , X. Weng , and Y. Liu . 2010 Structural modifications of the brain in acclimatization to high‐altitude. PLoS One 5:e11449. 20625426 10.1371/journal.pone.0011449 PMC2897842

[brb3512-bib-0063] Zhang, Z. , W. Liao , H. Chen , D. Mantini , J.‐R. Ding , Q. Xu , et al. 2011 Altered functional‐structural coupling of large‐scale brain networks in idiopathic generalized epilepsy. Brain 134:2912–2928. 21975588 10.1093/brain/awr223

[brb3512-bib-0064] Zhang, H. , J. Lin , Y. Sun , Y. Huang , H. Ye , X. Wang , et al. 2012 Compromised white matter microstructural integrity after mountain climbing: evidence from diffusion tensor imaging. High Alt. Med. Biol. 13:118–125. 22724615 10.1089/ham.2011.1073

[brb3512-bib-0065] Zhang, J. , H. Zhang , J. Li , J. Chen , Q. Han , J. Lin , et al. 2013 Adaptive modulation of adult brain gray and white matter to high altitude: structural MRI studies. PLoS One 8:e68621. 23874692 10.1371/journal.pone.0068621 PMC3712920

[brb3512-bib-0066] Zhu, L. , T. Zhao , H. Li , H. Zhao , L. Wu , A. Ding , et al. 2005 Neurogenesis in the adult rat brain after intermittent hypoxia. Brain Res. 1055:1–6. 16098951 10.1016/j.brainres.2005.04.075

[brb3512-bib-0101] Zubieta‐Calleja, G. R. , P. E. Paulev , L. Zubieta‐Calleja , and G. Zubieta‐Castillo . 2007 Altitude adaptation through hematocrit changes. J. Physiol. Phamacol. 58:811–818. 18204195

[brb3512-bib-0067] Zuo, X. N. , C. Kelly , A. Di Martino , M. Mennes , D. S. Margulies , S. Bangaru , et al. 2010 Growing together and growing apart: regional and sex differences in the lifespan developmental trajectories of functional homotopy. J. Neurosci. 30:15034–15043. 21068309 10.1523/JNEUROSCI.2612-10.2010 PMC2997358

